# Liver and Gastrointestinal Involvement in Patients With COVID-19: A Retrospective Study

**DOI:** 10.7759/cureus.24580

**Published:** 2022-04-29

**Authors:** Mahdi E Aljaroudi, Sulaiman K Alghamdi, Balqis A Al Salman, Mohammed J Alabdulghani

**Affiliations:** 1 Gastroenterology and Hepatology, Dammam Medical Complex, Dammam, SAU; 2 Gastroenterology and Hepatology, King Fahad General Hospital Hofuf, Al Ahssa, SAU; 3 Gastroenterology and Hepatology, Al-Qatif Central Hospital, Qatif, SAU

**Keywords:** ksa:kingdom of saudi arabia, retrospective study, gastro-intestinal tract complications, liver injury, covid-19

## Abstract

Background

Coronavirus disease 2019 (COVID-19) classically presents as a respiratory illness with fever, dry cough, and dyspnea on exertion. Along with respiratory signs and symptoms, gastrointestinal (GI) manifestations and liver injury have been recognized during the progression of the disease. This study aimed to determine the prevalence of GI symptoms and hepatic injury during COVID-19 infections and their consequences on the outcome of the disease.

Methodology

We conducted a retrospective survey of 715 participants age 16 or older diagnosed with COVID-19 and reported GI and hepatic manifestations in the Dammam Medical Complex in Dammam, Eastern Province, Saudi Arabia, from March 1, 2020, to May 31, 2020. We recorded clinical manifestations, laboratory test results, patient demographics, comorbidities, and treatments.

Results

The mean age of the study population was 46 years (88% were male, 12% were female), and 80% were non-Saudi. While most patients recovered and were discharged (n=603, 84.62%), 100 (13.99%) died due to COVID-19. Type 2 diabetes was present in 182 patients (79%) discharged and 45 patients (21%) who died. Hypertension was present in 26 (67%) discharged and 158 patients (81%) who died. Cardiovascular disease was present in 26 patients (67%) discharged and 13 (33%) who died. Chronic kidney disease was found in 11 patients (61%) discharged and six (33%) who died. Diarrhea was present in 11% of patients, nausea in 8%, and vomiting in 9% of patients. Twenty percent of patients had at least one GI symptom. Only 10% of those who died had GI symptoms, while 88% of those discharged had GI symptoms. Serum levels of alanine aminotransferase, aspartate aminotransferase, total bilirubin, alkaline phosphatase, and γ-glutamyl transpeptidase were generally higher in the patients who died than in those who were discharged.

Conclusions

We noted an increase in at least one liver enzyme with no clinically significant acute liver injury or cases of acute liver failure as sequelae of COVID-19. However, the presence of injury at the time of admission resulted in a significantly higher mortality rate. Only a small number of patients infected with COVID-19 exhibited GI manifestations. The etiology of severe acute respiratory syndrome coronavirus 2-related GI involvement is due to multiple factors. It is not yet fully understood if GI manifestations are clinical signs of high viral loads or another physiological process. The clinical manifestation and laboratory test results indicate that COVID-19 impacts the hepatic system and GI tract, indicating that COVID-19 infection may risk liver and GI tract injury.

## Introduction

Coronavirus disease 2019 (COVID-19) is a respiratory illness caused by infection of the severe acute respiratory syndrome coronavirus 2 (SARS-CoV-2) and is especially dangerous for patients with chronic respiratory conditions or compromised immune systems [1-3]. Researchers have studied the various clinical manifestations of COVID-19, such as asymptomatic infection, fatal signs, and severe conditions requiring endotracheal intubation [4]. The Chinese Center for Disease Control and Prevention reported that 80% of patients have mild signs and symptoms and do not need hospital admission, but 15% of patients develop moderate symptoms such as pneumonia and respiratory dysfunction and need hospital admission [5]. Only 5% of COVID-19 patients develop severe lung dysfunction, extrapulmonary organ failure, and shock and need admission to the intensive care unit (ICU) for endotracheal intubation [5]. Patients with severe COVID-19 admitted to the ICU have a 30% to 70% mortality rate [6].

SARS-CoV-2 attaches to the angiotensin-converting enzyme II (ACE II) receptors extensively expressed in gastric, duodenal, and rectal epithelia. Thus, gastrointestinal (GI) infection with SARS-CoV-2 is feasible, and fecal-oral transmission is possible. In addition, ACE II receptors can also be expressed in hepatic cholangiocytes and hepatocytes, potentially enabling direct infection of hepatic cells.

A small proportion of COVID-19 patients may present with abnormal liver chemistry and GI concerns, including loss of appetite, nausea, vomiting, abdominal pain, and diarrhea [7]. However, reports of GI and hepatic manifestations of COVID-19 vary significantly in the literature. For example, diarrhea is reported in 1% to 36% of COVID-19 patients [8,9]. Recent reports suggest that GI and hepatic manifestations of COVID-19 are higher than initially reported, particularly in Western populations [10]. This study aimed to investigate GI and hepatic complications due to COVID-19 in adults.

## Materials and methods

Study design

We conducted a retrospective cross-sectional survey of all consecutive patients diagnosed with COVID-19 treated at the Dammam Medical Complex in Dammam, Eastern Province, Saudi Arabia, from March 1, 2020, to May 31, 2020. Patient diagnoses of COVID-19 were confirmed via polymerase chain reaction testing. Patients were included if they were older than 15 years and underwent a complete panel of routine blood work, including complete blood count, blood biochemistry, renal and liver function tests, and blood coagulation function tests. The study also included COVID-19 patients with type 2 diabetes (T2D), hypertension (HTN), and cardiac diseases. Patients were excluded if they had hepatitis B or C viral coinfections or were treated with hepatotoxic medications (e.g., amiodarone, tamoxifen, methotrexate, steroids, or hydroxychloroquine). Patients taking herbal remedies or those who consume more than 20 g of alcohol per day were excluded. We also excluded patients with genetic or metabolic liver disease or other underlying liver diseases, such as autoimmune hepatitis, primary biliary cirrhosis, hepatoma, and decompensated cirrhosis.

Ethics approval

The study design was approved by the Dammam Medical Complex Institutional Review Board (IRB-D-2020-11), and the study complied with the Declaration of Helsinki.

Data collection and management

We collected data from patient medical records (physical and electronic files). We gathered patient baseline epidemiological and demographic data, clinical characteristics, symptoms at presentation (including GI and respiratory symptoms), laboratory data, treatment regimens, whether oxygen was required, and outcome measures (e.g., if they were discharged, died, or transferred to another health care facility or quarantine site). Data collection was as thorough as possible through chart review and communication with other medical staff to fill in the missing data. We defined GI involvement as the presence of abdominal pain, nausea, vomiting, diarrhea, or loss of appetite. Patients were considered to have a liver injury at presentation if they had elevated liver markers such as alanine aminotransferase (ALT), aspartate aminotransferase (AST), total bilirubin, or alkaline phosphatase (ALP). Patients without complete data were excluded from the analysis.

Statistical analysis

Analytic data were presented as means ± standard deviation (SD) for normally distributed continuous variables and as medians with interquartile ranges for non-normally distributed data. Categorical variables were presented as percentages. We also evaluated whether the measurements were outside the target range based on the reference ranges used in our institution for laboratory results. All statistical analyses were performed using IBM SPSS Statistics for Windows, Version 25.0 (IBM Corp., Armonk, NY, USA). The student’s t test was used to test two independent samples; analysis of variance or the Kruskal-Wallis rank-sum test was used to compare multiple groups. The Chi-square test compared count data, and a two-tailed p<0.05 was considered statistically significant.

## Results

Demographic characteristics

**Figure 1:**
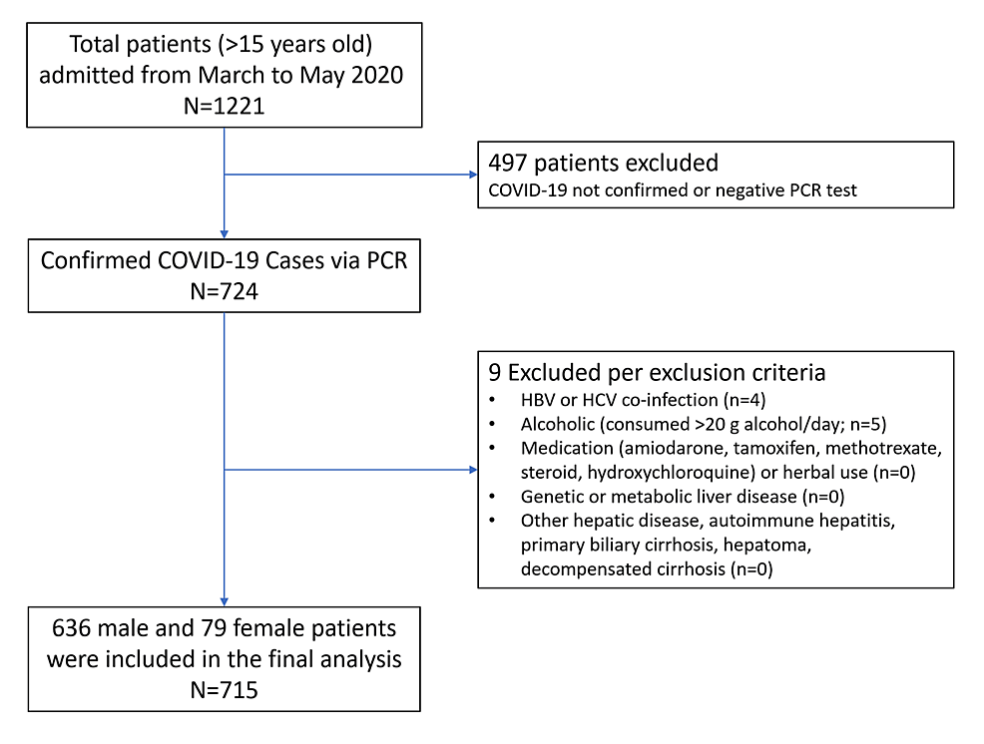
Study flow diagram HBV; hepatitis B virus, HCV; hepatitis C virus

From March 1 to May 31, 2020, 1212 patients were admitted to the Dammam Medical Complex in Dammam, Eastern Province, Saudi Arabia. Of these, 724 patients were diagnosed with COVID-19 [11]. Nine of the 724 patients were excluded (four due to hepatitis B virus (HBV) or hepatitis C virus (HCV) coinfection and five due to alcoholism) for a total population of 715 patients. Figure 1 presents the flow of patient selection for inclusion.

**Figure 2:**
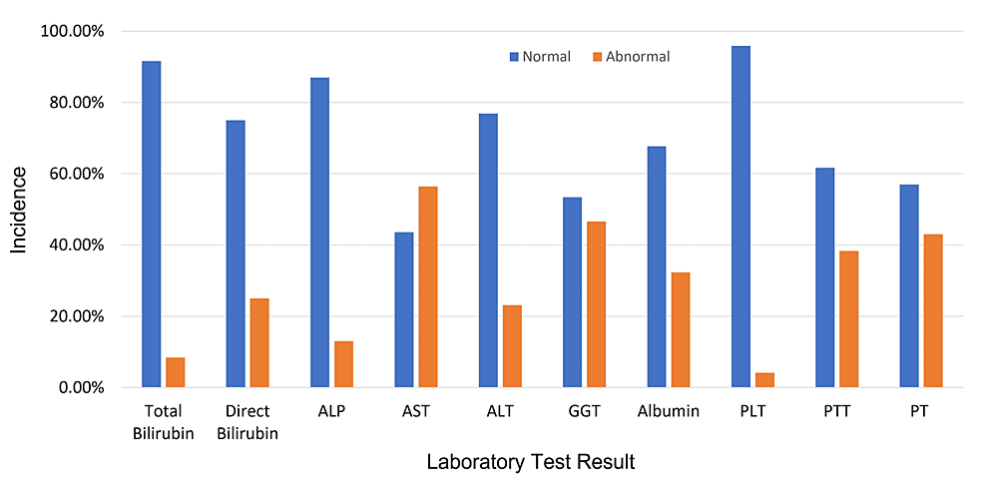
Laboratory and liver biochemical findings for all patients presenting with COVID-19 COVID-19, coronavirus disease 2019; ALP, alkaline phosphatase; AST, aspartate aminotransferase; ALT, alanine aminotransferase; GGT, gamma-glutamyl transferase; PLT, platelets; PTT, partial thromboplastin time; PT, prothrombin time

The mean patient age was 46 years; 88% were male, 12% were female. Most patients (80%) were non-Saudi. Most patients (n=603, 84.62%) recovered from COVID-19 and were discharged, and 100 (13.99%) died due to COVID-19. As shown in Figure 2, the mean age of patients who died was 52 years (F=16.051, p=0.000), significantly older than the mean age of discharged patients (45 years; range, 15 to 100). Among the 94 patients over age 60, 28 died (29%), and 64 were discharged (68%). There was a larger proportion of males in the group who died (n=93, 93%) than survivors discharged (n=533, 74%; F=3.357; p=0.187), and a small number of patients had a history of cigarette smoking, which was statistically insignificant (Table 1).

**Table 1:** Demographic and clinical findings of patients presenting with COVID-19 COVID-19, coronavirus disease 2010; SD, standard deviation; T2D, type 2 diabetes: HTN, hypertension; CKD, chronic kidney disease

Variable		Total	Discharge	Transfer to Another Facility	Death	P-Value
Male, n (%)		636 (88.9%)	533 (88.0%)	10 (100.0%)	93 (93.0%)	0.339
Female, n (%)		79 (11.1%)	72 (12.0%)	0 (0.0%)	7 (7.0%)	
Age in years, mean ± SD		46.5 ± 12.2	45.4 ± 11.7	49.8±12.4	52.6 ± 13.0	< .001>
Preexisting Comorbidities						
T2D, n	Yes	231 (32.3%)	182 (30.1%)	4 (40.0%)	45 (45.0%)	0.01
	No	484 (67.6%)	423 (69.9%)	6 (60.0%)	55 (55.0%)	
HTN, n	Yes	195 (27.2%)	158 (26.1%)	2 (20.0%)	35 (35.0%)	0.158
	No	520 (72.2%)	447 (74.2%)	8 (80.0%)	65 (65.0%)	
Diarrhea, n	Yes	81 (11.3%)	72 (11.9%)	1 (10.0%)	8 (8.0%)	0.517
	No	634 (88.7%)	533 (88.1%)	9 (90.0%)	92 (92.0%)	
Nausea, n	Yes	60 (8.4%)	53 (8.8%)	1 (10.0%)	6 (6.0%)	0.634
	No	655 (91.6%)	552 (91.2%)	9 (90.0%)	94 (94.0%)	
Vomiting, n	Yes	69 (9.6%)	62 (8.7%)	0 (0.0%)	7 (7.0%)	0.346
	No	646 (90.3%)	543 (89.7%)	10 (100.0%)	93 (93.0%)	
CKD, n	Yes	18 (2.5%)	11 (1.8%)	1 (10.0%)	6 (6.0%)	0.015
	No	697 (97.5%)	594 (98.2%)	9 (90.0%)	94 (94.0%)	
Cardiovascular disease, n	Yes	39 (5.4%)	26 (4.3%)	0 (0.0%)	13 (13.0%)	0.011
	No	676 (94.5%)	579 (95.7%)	10 (100.0%)	87 (87.0%)	
Required ventilator support, n	Yes	124 (17.3%)	25 (4.1%)	9 (90.0%)	90 (90.0%)	< .001>
	No	591 (82.6%)	580 (95.9%)	1 (10.0%)	10 (10.0%)	
Gastrointestinal symptoms, n	Yes	146 (20.4%)	129 (21.3%)	2 (20.0%)	15 (15.0%)	0.348
	No	569 (79.5%)	476(78.7%)	8 (80.0%)	85 (85.0%)	
Total, n		715	605	10	100	

Clinical manifestation

T2D was present in 182 patients (79%) who were discharged and 45 (21%) who died (F=9.006; p=0.01). HTN was noted in 158 patients (81%) who were discharged and 25 (19%) who died (F=3.685; p=0.158). Cardiovascular disease was found in 26 patients (67%) discharged home and 13 (33%) who died (F=8.030; p=0.011). Chronic kidney disease was present in 11 patients (61%) who were discharged and six (33%) who died (F=8.429; p=0.015). At presentation, 11% of patients had diarrhea, 8% had nausea, and 9% experienced vomiting. Twenty percent of COVID-19 patients experienced at least one GI symptom, and GI symptoms were less common in those who died (n=15, 10%) than in those who were discharged (n=129, 88%; F=2.112; p=0.348) (Table 1).

The mean time from admission to death was seven days (range, zero to 33 days), and the mean time from admission to discharge was eight days (range, zero to 46 days; F=1.357; p=0.258). Of those that died, 100 (24%) required oxygen on admission, which was a significantly lower proportion than the 305 patients (73%) who required oxygen and were later discharged (F=90.567; p<.001). Of those patients who died, 90 (72%) required ventilatory support during their hospitalization compared with 25 (20%) patients who were discharged after needing ventilatory support (F=478.74; p<.001) (Figure 2).

More patients who died required vasopressor support (n=67, 76%) than those discharged (n=13, 14%). Use of steroids, antibiotics, and anti-viral medication was higher among the deceased at 51%, 100%, and 76%, respectively, than those discharged (6%, 85%, and 29%, respectively).

Laboratory tests

On admission, patients had a mean ALT of 48.84 U/L (target range: 0-65 U/L), mean AST of 54 U/L (target range: 0-37 U/L), mean total bilirubin of 0.66 mg/dL (target range: 0-0.6 mg/dL) and mean ALP of 76.8 U/L (target range: 44-147 U/L). More than half of the patients in our study (56%) had biochemical evidence of liver injury with a minimum of one elevated liver enzyme at the time of presentation [7].

Seventy-six patients who died (76%) and 322 who were discharged (53%) had elevated AST levels (>37 U/L; F=18.136, p<.001) [12]. Albumin levels were substantially lower in patients who died than in those who were discharged. Hypoalbuminemia (i.e., albumin <3.4 g/dL) was observed in 56 patients who died (56%) and 167 who were discharged (28%). Serum levels of blood urea nitrogen, creatinine, and potassium were significantly higher in patients who died than in those who were discharged. Prothrombin time was longer in patients who died (45%) than in those who were discharged (43%). In contrast, activated partial thromboplastin time was statistically different: 54% higher in those who died and 35% higher in those discharged (F=12.876, p=0.002). Platelet counts were lower (3%) in patients who died than in those discharged (4%; F=1.2, p=0.55).

We found a significant difference in laboratory results between the deceased and discharged patients (Table 2). Serum levels of ALT, AST, total bilirubin, ALP, and γ-glutamyl transpeptidase were generally higher in the patients who died than in those who were discharged.

**Table 2:** Laboratory findings of patients presenting with COVID-19 ^a ^Based on laboratory references used at the Dammam Medical Complex. *Significant difference (p< 0.05). **High Significant difference (p<.001) COVID-19, coronavirus disease 2019; ANOVA, analysis of variance; ALP, alkaline phosphatase; AST, aspartate aminotransferase; ALT, alanine aminotransferase; GGT, gamma-glutamyl transferase; PLT, platelets; PTT, partial thromboplastin time; PT, prothrombin time.

Test	Reference Range^a^	Discharge, n (mean)	Transfer to Another Facility, n (mean)	Death, n (mean)	ANOVA F/ Significance
Total bilirubin	0–1 (mg/dL)	593 (0.63 mg/dL)	10 (0.68 mg/dL)	98 (0.67 mg/dL)	0.23 / 0.795
Direct bilirubin	0–0.3 (mg/dL)	591 (0.66 mg/dL)	10 (0.3 mg/dL)	98 (0.6 mg/dL 1)	0.26 / 0.769
ALP	44–147 (IU/L)	594 (73 IU/L)	10 (84.1 IU/L)	99 (99.2 IU/L)	12.32 / .001
AST	0–37 (U/L)	604 (52.1 U/L)	10 (54.9 U/L)	100 (66.1 U/L)	4.29 / 0.014*
ALT	0–65 (U/L)	602 (48.7 U/L)	10 (47.3 U/L)	99 (49.7 U/L)	0.038 / 0.963
GGT	5–55 (U/L)	586 (78.7 U/L)	10 (133.6 U/L)	97 (106.19 U/L)	4.77 / 0.009*
Albumin	3.4–5 (g/dL)	596 (3.65 g/dL)	10 (3.33 g/dL)	99 (3.14 g/dL)	38.97 / .001
PLT	150–450 (×10^9^/L)	602 (245.9×10^9^/L)	10 (288.9×10^9^/L)	100 (230×10^9^/L)	1.84 / 0.159
PTT	25–35 s	515 (32.98 s)	10 (35.46 s)	94 (36.48 s)	10.05 / .001
PT	11–13.5 s	516 (11.81 s)	10 (12.12 s)	95 (12.42 s)	5.889 / 0.003*

## Discussion

By the end of May 2020, more than six million people worldwide had been infected with SARS-CoV-2. By that time, more than 85,260 cases had been confirmed in Saudi Arabia [13]. Like most other countries, Saudi Arabia responded proactively to prevent the spread of SARS-COV-2 [14,15]. Despite experiencing pandemic peaks, Saudi Arabia shortened the quarantine period for individuals and families via early preventive measures [16]. The number of confirmed infections and infection-related mortalities has decreased compared with other countries. Compared to its effects in the Middle East and other regions, quarantine did not impose significant restrictions on travel or daily life in Saudi Arabia. However, a series of extreme preventive measures did prevent pilgrims from visiting the two holy cities of Mecca and Medina. Moreover, King Salman bin Abdulaziz ordered all governmental and private health facilities inside the Kingdom to provide free COVID-19 treatments [17].

In our study, only a few COVID-19 patients presented GI symptoms, which did not support a significant correlation between COVID-19 and GI manifestations [18,19]. An increase in at least one liver enzyme was observed in 56% of cases, which was similar to other reports [20]. We observed a substantial increase in the incidence of liver injury in those admitted with severe pneumonia before they underwent multi-organ dysfunction during hospitalization in 76% of the dead cases, which supports the literature that concludes the presence of liver injury at admission resulted in a significantly higher mortality rate. The liver injury accompanying COVID-19 may be due to direct viral infection of the hepatocytes. The pathophysiology of liver injury observed in COVID-19 was heterogeneous and due to multiple factors, including an indirect reaction of the systemic inflammatory response, which resulted in significant immune hyperactivity, cytokine pathway activation, and compromised intravascular hemostasis.

Tian et al. investigated the hepatic complications among COVID-19 patients with respiratory tract infection (RTI). They found a liver injury condition in participants with severe RTI occurred via significant systemic inflammation, cytokine storm, and ischemia-reperfusion injury. Patients in their study had liver biochemical test results outside of reference ranges, but no patients experienced liver failure [21]. Cha et al. conducted a comprehensive review and found that some COVID-19 patients have GI complications such as vomiting, nausea, diarrhea, abdominal discomfort, and liver biochemical functions outside of reference ranges, such as elevated serum AST, ALT, and total bilirubin levels [22]. Omrani-Nava et al. also reported liver enzyme levels outside of reference ranges for COVID-19 patients but found no cases of liver damage. They attributed this to systematic inflammation due to COVID-19 infections [23].

Study limitation

Because this was a retrospective single-center study with limited data from medical records, these findings may not be widely applicable as this study was conducted early in the pandemic when experience with COVID-19 treatment was limited. It is unknown if these results should be presented as a time-varying continuation of demographics and clinical data. Also, because the study period was short, the outcome of hospitalization could not be further evaluated.

## Conclusions

The goal of this study was to investigate GI and hepatic complications due to COVID-19 in adults. The clinical manifestation and laboratory tests indicate COVID-19 can impact the hepatic system and GI tract, but only a small number of patients infected with COVID-19 exhibited GI symptoms, while more than half of the patients had at least one abnormal liver enzyme as a hepatic manifestation in our retrospective study. It is not fully understood if GI manifestations are clinical signs of higher viral loads or another physiological process, but physicians should consider the presence of these symptoms when forming their initial clinical and management plans for these patients.
